# Crisis preparedness exercise on food and feed safety for IPA beneficiaries 2024

**DOI:** 10.2903/sp.efsa.2024.e221001

**Published:** 2024-10-18

**Authors:** European Food Safety Authority (EFSA), Alberto Mancuso (Opera srl)

**Keywords:** crisis preparedness, simulation exercise, training, biological contamination, IPA beneficiaries

## Abstract

The European Food Safety Authority (EFSA) within the framework of the Preparatory measures for the participation of IPA beneficiaries in the European Food Safety Authority 2023–2026 will organize a total of 3 crisis preparedness training events for the IPA countries (Albania, Bosnia and Herzegovina, Kosovo,* Montenegro, North Macedonia, Serbia, Türkiye). For 2024, EFSA requested to organise a simulation exercise focused on a multi‐country incident related to biological risks in products of animal origin*.* The overall objectives of the training were: 1) to increase IPA countries' knowledge and understanding of crisis handling concepts and of EFSA's crisis handling procedures; 2) to improve preparedness and response planning for crisis situations in the domain of biological hazards; 3) to improve coherence, interoperability and coordination, to be prepared for communication and decision‐making challenges in crisis situations. Working with the contractor Opera srl, EFSA organised a 2‐day training on 26–27 June 2024 in Sarajevo (Bosnia and Herzegovina), attended by 24 participants (3 online) from 7 IPA countries, 2 representatives from EFSA and 1 representative from the hosting competent authority from Bosnia and Herzegovina. Participants were briefed on the IPA crisis preparedness training scheme, EFSA's crisis handling procedures, principles of crisis communication (remote mode), EFSA – ECDC Rapid Outbreak Assessment (remote mode), food safety risk assessment structures in Bosnia and Herzegovina and Serbia. Most of the workshop was dedicated to the simulation exercise, that was organized in a tabletop format, with injects describing an evolving food safety incident and a debriefing after each inject. During the exercise, 1 audio‐conference meeting and a TV debate were simulated. The exercise was concluded with a brainstorming session between the tutors and participants. Three recommendations were done to improve the organization of future events. Six recommendations were given for the improvement of risk assessment, risk management and risk communication in IPA countries. The objectives were achieved, based on the results of the knowledge test and on the feedback provided by participants in the course evaluation questionnaire.

* This designation is without prejudice to positions on status, and is in line with UNSCR 1244 and the ICJ Opinion on the Kosovo Declaration of Independence

**Disclaimer:** The views or positions expressed in this publication do not necessarily represent in legal terms the official position of the European Food Safety Authority (EFSA). EFSA assumes no responsibility or liability for any errors or inaccuracies that may appear.

**Acknowledgements:**

For their support in preparing and delivering the event: Victoria Villamar (EFSA), Matthew Ramon (EFSA), Kerstin Gross-Helmert (EFSA), Sanja Ilieva (EFSA), Georgia Gkrintzali (EFSA) and Dalibor Vidacak (Food Safety Agency of Bosnia and Herzegovina).

For their development and delivery of presentations: Eleonora Sarno (EFSA) and Giovanni Mancarella (EFSA).

**Suggested citation:** EFSA (European Food Safety Authority), 2024. Crisis preparedness exercise on food and feed for IPA beneficiaries 2024. EFSA Supporting publication 2024:e221001. 21 pp. doi:10.2903/sp.efsa.2024.e221001

**ISSN:** 2397-8325

Reproduction is authorised provided the source is acknowledged.

**Map disclaimer:** The designations employed and the presentation of material on any maps included in this scientific output do not imply the expression of any opinion whatsoever on the part of the European Food Safety Authority concerning the legal status of any country, territory, city or area or of its authorities, or concerning the delimitation of its frontiers or boundaries.

## Summary

The European Food Safety Authority (EFSA), in collaboration with Opera srl and the Food Safety Agency of Bosnia and Herzegovina, organized a 2-day crisis preparedness training workshop for the IPA countries (Albania, Bosnia and Herzegovina, Kosovo[1], Montenegro, North Macedonia, Serbia, Türkiye). The event took place on 26 and 27 June 2024 in Sarajevo, Bosnia and Herzegovina.

The overall objectives of the training were: 1) to increase IPA countries' knowledge and understanding of crisis handling concepts and of EFSA's crisis handling procedures; 2) to improve preparedness and response planning for crisis situations in the domain of biological hazards; 3) to improve coherence, interoperability and coordination, to be prepared for communication and decision-making challenges in crisis situations.

Each IPA country appointed 4 participants, with a background in risk assessment or in risk management or in risk communication. The workshop was attended by 24 participants (3 online) from 7 IPA countries, 1 coordinator and 3 tutors (2 online) from EFSA and 1 organizer from Bosnia and Herzegovina.

For 2024, EFSA requested to organise a simulation exercise focused on a multi-country incident related to biological risks in products of animal origin. It was agreed to focus on a prolonged multi-country outbreak of listeriosis cases caused by *Listeria monocytogenes* sequence type 8, that was identified through Whole Genome Sequencing (WGS) in different EU countries. The temporal distribution of cases indicated a prolonged intermittent common-source outbreak taking place in different EU Member States. Traceability information of the contaminated products referred to a processing Company as the single common manufacturer of contaminated fish products.

Before the simulation exercise, participants were briefed by EFSA colleagues on the IPA crisis preparedness training scheme, EFSA's crisis response procedures, crisis communication during a multi-country food/feed safety incident (remote mode), WGS in Rapid Outbreak Assessment (remote mode). Two participants, respectively from Bosnia and Herzegovina and Serbia, presented an overview of the National food safety risk assessment structures in their countries.

The exercise was organized in a tabletop format. Three Working Tables were appointed, each of them composed of participants from different countries. Participants were provided with a series of subsequent injects, describing an evolving food safety incident. After each inject, participants were asked to assess the documents provided, evaluate the situation and answer to specific questions in written form. Multi-disciplinary group working was required.

The simulation exercise was organized in 7 injects and a final session focused on conclusions and debriefing. During the simulation exercise, 1 audio-conference meeting and a TV debate were simulated.

[1] *This designation is without prejudice to positions on status, and is in line with UNSCR 1244 and the ICJ Opinion on the Kosovo Declaration of Independence*

In the final session, participants were engaged in brainstorming exercise. Three recommendations were made concerning the organization of future events. Six recommendations were made for the improvement of risk assessment, management and communication in IPA countries.

The objectives were achieved, based on the results of the knowledge test and on the feedback provided by participants in the course evaluation questionnaire.

## Table of contents

Abstract ........ 1
Summary ........ 3
Table of contents ........ 5
Introduction ........ 6
Detailed description of the training ........ 7
Training dates, location and participants ........ 7
Method ........ 7
Means of preparation of participants (reading list) ........ 8
Training materials and documents ........ 9
Training program ........ 9
Organisation of the simulation exercise ........ 11
Topic of the scenario: case study on the EU food safety system ........ 12
Assessment of the training ........ 12
Measures of workshop success ........ 12
Proficiency: knowledge gain of participants (“test”) ........ 13
Quality of the workshop and participants satisfaction ........ 15
Comments ........ 16
Conclusion ........ 17
Recommendations ........ 17
Recommendations for future crisis preparedness activities ........ 17
Recommendations for IPA countries ........ 18
References ........ 20
Abbreviations ........ 21

## Introduction

In the framework of the Contract No NP/EFSA/ENREL/2023/03 "Multiannual crisis preparedness exercises on food and feed safety in IPA beneficiaries", signed between EFSA on the one part, and Opera S.r.l., Limited liability company, Viale Parioli, n. 96, 00197, Roma (Italy) on the other part, the Order Form number 01 was received from Opera on 27/3/2024.

The objective of the assignment was to develop and organise a crisis-preparedness workshop on food and feed safety in the IPA countries Albania, Bosnia and Herzegovina, Kosovo[2], Montenegro, North Macedonia, Serbia and Türkiye. The overall objectives of the training were to:

- increase IPA countries' knowledge and understanding of general crisis handling concepts and specifically of EFSA's crisis handling procedures;
- improve preparedness and response planning for crisis situations in the domain of biological hazards through testing procedures in place at national (contingency plans) and EU level (e.g. the crisis general plan) and crisis handling procedures, helping IPA countries address crisis-preparedness needs and reduce time for action;
- improve coherence, interoperability and coordination at local level and between the local, national, regional and EU levels, to be prepared for communication and decision-making challenges in crisis situations.

The aim of the assignment was to organise and execute a training workshop, which included:

- Developing a training plan and training materials;
- Collecting feedback from workshop participants and measuring workshop success;
- Advising EFSA and the IPA countries by making recommendations on areas that need further improvement for crisis-preparedness.

The expected outcomes of the training were:

- developed/enhanced urgent response capacities in risk assessment and risk communication;
- improved incident response collaboration between IPA national coordinating institutions involved during crisis situations within EFSA's remit, and between IPA countries and EFSA;
- improved crisis-preparedness procedures.

According to the Order Form n. 01 of 27/03/2024, the Contractor prepared a detailed Training Workshop Plan, including the following elements:

- topic and an outline of the content of the crisis preparedness workshop scenarios,
- expertise to be used for the training,

[2] *This designation is without prejudice to positions on status, and is in line with UNSCR 1244 and the ICJ Opinion on the Kosovo Declaration of Independence*

- indication of training materials, documents, techniques to be used during the training,
- means of preparation of participants (e.g. reading list and access to materials), if relevant,
- means of evaluating the knowledge gain of participants and the quality of the workshop,
- measures of workshop success.

On the basis of the contents of the Training Workshop Plan, a 2-day crisis preparedness training workshop for the IPA countries was organised on 26 and 27 June 2024 in Sarajevo (BiH).

## Detailed description of the training

### Training dates, location and participants

The workshop was held 26th and 27th June 2024 at the Center for interdisciplinary studies, University of Sarajevo, Bosnia and Herzegovina. The venue was proposed by the Food Safety Agency of Bosnia and Herzegovina. EFSA Focal point observer for Bosnia and Herzegovina provided full support before and during the workshop, and this was crucial for the success of the event.

The expected number of participants was 28 from IPA countries (Albania, Bosnia and Herzegovina, Kosovo[3], Montenegro, North Macedonia, Serbia and Türkiye). A total of 24 participants attended the training (3 of them attended online). Participants were nominated in cooperation and communication between EFSA and the national coordinating institutions in the IPA countries.

### Method

The workshop was carried out in lessons/discussions during which the exchange of knowledge and the comparison between the experiences gained by participants in their professional activities was encouraged.

A large part of the training course was dedicated to practical exercises, at the aim to assess the acquired knowledge of the participants regarding the topics, and to provide them with practical tools to be used during daily work activity.

This practical training was particularly appreciated by the participants giving them the opportunity to compare and discuss their experience with colleagues coming from the other IPA countries.

The workshop was chaired by Ms Sanja Ilieva from EFSA, and co-chaired by Mr Alberto Mancuso from Opera.

[3] *This designation is without prejudice to positions on status, and is in line with UNSCR 1244 and the ICJ Opinion on the Kosovo Declaration of Independence*

The following experts had role of facilitators for the whole duration of the workshop:

- Georgia Gkrintzali, EFSA
- Alberto Mancuso, Opera
- Ana Canals, Opera
- Mats Lindblad, Opera
- Stefano Pongolini, Opera
- Dalibor Vidacak, Food Safety Agency of Bosnia and Herzegovina

### Means of preparation of participants (reading list)

The proposed design of the training was sent to all participants 2 weeks before the training. The document highlighted the main topics of the workshop and contained references to EFSA procedures and relevant EU Regulations. The following documents were made available to participants, with the objective to get participants acquainted with the topics that were to be presented at the workshop.

*Supporting documentation*

- Presentation: EU legal framework on crisis management coordination and response in the food and feed sectors.
- Regulation (EU) 2022/2371 of the European Parliament and of the Council on serious cross-border threats to health and repealing Decision No 1082/2013/EU.
- Commission Implementing Decision (EU) 2019/300 establishing a general plan for crisis management in the field of the safety of food and feed.
- Joint ECDC–EFSA Rapid Outbreak Assessment. Prolonged multi-country outbreak of *Listeria monocytogenes* ST1607 linked to smoked salmon products – 25 April 2024.
- EFSA procedures for crisis response.
- EFSA. Best practice for crisis communicators. How to communicate during food or feed safety incidents. 2016.

The participants also received additional reading literature for an in-depth analysis of the topics of the workshop, to be used as an optional reading material.

*Additional documents*

- Regulation (EC) 178/2002 of the European Parliament and of the Council laying down the general principles and requirements of food law, establishing the European Food Safety Authority and laying down procedures in matters of food safety, as amended by Regulation (EU) 2019/1381 of the European Parliament and of the Council on the transparency and sustainability of the EU risk assessment in the food chain.
- Directive 2003/99/EC on the monitoring of zoonoses and zoonotic agents, as amended.
- Regulation (EU) 2017/625 of the European Parliament and of the Council on official controls and other official activities performed to ensure the application of food and feed law, rules on animal health and welfare, plant health and plant protection products.

- EFSA and ECDC. The European Union One Health 2022 Zoonoses Report. EFSA Journal, Volume 21, Issue 12. November 2023.
- ECDC EFSA. EFSA and ECDC technical report on the collection and analysis of whole genome sequencing data from food-borne pathogens and other relevant microorganisms isolated from human, animal, food, feed and food/feed environmental samples in the joint ECDC–EFSA molecular typing database. EFSA supporting publications, Volume 16, Issue 5, May 2019
- EFSA. When food is cooking up a storm. Proven recipes for risk communications. May 2017.
- RASFF Standard operating procedures (SOPs) and other relevant SOPs (e.g. EWRS).

### Training materials and documents

Participants received the following training material and documents in electronic format:

- Supporting documentation (Reading list, see previous chapter)
- Copy of all presentations
- Agenda for the exercise
- Copies of all injects (shared in real time during the exercise)

At the end of the course, each participant received a certificate of attendance.

### Training program

The agenda covered two full days of training, with a structure that is summarized in the following paragraphs.

The workshop started on 26th of June at 08:30. Participants were welcomed by Ms. Sanja Ilieva from EFSA and the facilitators. As an icebreaker, participants were asked to briefly introduce themselves and were invited to proactively take part in the discussions and to express openly their opinion on their expectations from the training. A presentation on the collaboration between EFSA and IPA countries was delivered by Ms. Sanja Ilieva (EFSA) and Mr. Dalibor Vidacak (Food Safety Agency of Bosnia and Herzegovina); the presentation also covered information on the capacity building initiatives covered with the current IPA Action and the relevant objectives and outcomes of each one of them. To better understand the national structures in the IPA countries, participants from Bosnia and Herzegovina and from Serbia were invited to deliver presentations on the organization set up on the national food safety systems. In addition to the presentation of the national food safety system, the participants from Serbia also presented a real case of food incident in their country and how the involved authorities have managed it.

*Presentation: EFSA's crisis response procedures*

With this presentation, that was delivered by Dr. Georgia Gkrintzali from EFSA, participants were provided with information on EFSA's crisis handling procedure, to create a common background of knowledge before starting the simulation exercise. The core of this presentation was the publication "EFSA procedures for crisis response", that provides guidance on the practical arrangements necessary to ensure that EFSA responds effectively to incidents requiring urgent advice on food and feed safety. More specifically an outline of what is an emergency, why EFSA is involved, what is specific for Rapid Risk Assessment, a concise overview of EFSA's procedure for crisis response and the impact of Regulation (EU) 2022/2073 on serious cross border threats to health on EFSA's procedures were highlighted during this presentation.

*Presentation: Crisis communication during a multi-country food/feed safety incident*

This presentation was given online by Mr. Giovanni Mancarella from EFSA. Participants were introduced to main documents and guidelines on crisis communication developed by EFSA. More in detail, the presentation focused on the document "Best practice for crisis communicators - How to communicate during food or feed safety incidents", that was developed to encourage consistent best practice during incidents related to EFSA's mandate, with the aim to provide clear and practical recommendations for communicating with external audiences during a food or feed-related incident. The presentation also focused on the following: 1) definition of a crisis (immediate, emerging, sustained); 2) key considerations when it comes to communication during a crisis (e.g. transparency, successful responses ("Acknowledge, Apologise, Act" (AAA) vs unsuccessful responses ("Deny, Diminish, Deflect" (DDD); 3) definition of the concept of the "Golden hour" and drafting of an "holding statement"; 4) check-lists needed to prepare for communicating during a crisis.

*Presentation: EFSA-ECDC Rapid Outbreak Assessments (ROA)*

This presentation was given online by Dr. Eleonora Sarno from EFSA. Main concepts on the functioning of Joint EFSA-ECDC Rapid Outbreak Assessment (ROA) were provided to participants in combination with WGS contribution to this process. Joint EFSA-ECDC ROA is produced by ECDC, EFSA, DG SANTE (EC) and Member States (MS), when there is need to rapidly communicate about the risk assessment to MS and EC. Joint ROAs regards the main foodborne diseases and other new, emerging or rare foodborne bacterial (including their toxins or metabolites), viral and parasitic infections, which may require a joint ECDC-EFSA ROA (this is evaluated case-by-case).

*Simulation exercise – background, objectives and expected results: Exercise format*

Before the exercise, participants were briefed using a PowerPoint presentation.

In order to motivate the participants, it is important to clearly formulate and communicate the objectives of the exercise and explain why it is to be carried out. Before commencing the exercise, objectives and aims were clearly presented.

The need for an effective cooperation within and between Member States and IPA countries, between Member States, IPA countries and the EU institutions, and between risk assessors, risk managers and risk communicators with a multidisciplinary view, was pinpointed from the very beginning, as this was one of the leading principle of the exercise.

*Exercise debrief: Lessons identified, actions required to ensure lessons learned*

After the implementation of the simulation exercise, a debriefing session took place, focused on problems identified and actions required to ensure lessons learnt. Participants engaged in a brainstorming on how to improve preparedness, management and communication in case of foodborne outbreaks.

### Organisation of the simulation exercise

The Simulation Training Exercise focused on the challenge of responding to a multi-national food safety-public health incident.

The exercise was planned to simulate how an international food safety incident develops, step by step, and how different expertise can contribute to the assessment, management, and communication during a food safety incident. At the end of the exercise, participants should have acquired a clear understanding of the roles played by each subject involved, with the aim of strengthening collaboration and cooperation among the subjects involved in the assessment, management, and communication during a serious cross border foodborne incident.

The work was organised in a tabletop format. Three Working Tables were appointed, each of them composed by participants from different countries. This supported establishing contacts between CA staff of different countries and facilitated at least informal exchanges. Each country appointed 4 participants, with a background in risk assessment or in risk management or in risk communication.

Participants were provided with a series of subsequent injects, describing an evolving food safety incident. The same injects were submitted to all the Working Tables. Each inject included a number of questions, to be discussed and answered within the group in a specified time period. Each Working Table was asked to work on all the questions. Some injects included annexes with information on the developing incident.

After each inject, participants were asked to assess the documents provided, evaluate the situation and answer to specific questions in written form. To provide answers to those questions, participants were asked to work as a team where inputs could originate from the public health or food safety areas. Multi-disciplinary group working was required. Participants played a different role according to the inject: in some case they covered the position of local/regional authority, in other cases they represented the National authority of a Member State.

Debriefing after each inject consisted in a plenary discussion, coordinated and orientated by the facilitators, focused on the actions / communications adopted by countries and authorities involved. Spokespersons from one or more Working Table were asked to present the outcome of the work done.

Each facilitator was assigned to a Working Table, with the tasks to submit the injects and relevant annexes, to provide explanations where required and, more in general, to support the participants during the whole exercise.

The simulation exercise was organized in 7 injects and a final session focused on conclusions and debriefing. During the simulation exercise, 1 audio-conference meeting and a TV debate were simulated.

#### Topic of the scenario: case study on the EU food safety system

A multi-country incident was simulated, where several Member States and Third Countries were involved in the distribution of contaminated products originating from one Member State, with human cases appearing over Europe and necessitating rapid exchange of sensitive data across administrations, departments and participating countries and a coordinated response to the incident.

The simulation exercise was focused on biological risks in products of animal origin, more specifically on a prolonged multi-country outbreak of listeriosis cases caused by *Listeria monocytogenes* sequence type 8, that was identified through Whole Genome Sequencing (WGS) in different EU countries, with some patients who died due to, or with, the disease. The scenario was developed taking into account recent joint rapid outbreak assessment (ROAs) published by EFSA and ECDC.

The temporal distribution of cases indicated a prolonged intermittent common-source outbreak taking place in different EU Member States, with the source of contamination of the outbreak that could still be active. Most patients were found to have consumed mixed salads containing cold-smoked fish products during the incubation period.

*L. monocytogenes* food isolates, matching the human outbreak strain by WGS, were detected at wholesale and retail level in different EU Member States from different lots of cold smoked salmon. Traceability information of the contaminated lots pointed to a processing Company as the single common manufacturer of these fish products. Environmental investigations and food testing at the processing plant showed the presence of *L. monocytogenes* matching the outbreak strain in some samples found at the processing line and in some lots of the final product.

Findings suggested that the contamination with *L. monocytogenes* matching the outbreak strain might have happened through food contact surfaces shared by fresh and smoked/salted products, upstream of the crossing point of the different lines of production. The presence of *L. monocytogenes* matching the outbreak strain over several years in the fish products suggested the persistence of the microorganism at the company’s premises.

### Assessment of the training

#### Measures of workshop success

As defined in the workshop plan, measure of success made reference to evaluation of skills and to appreciation by participants, as follows:

- <u>Proficiency</u>: at least 80% of participants have successfully acquired knowledge. This was measured individually, and the test was considered successful if at least 6 out of 10 answers were answered correctly.
- <u>Participants satisfaction</u>: cumulative positive feedback from at least 80% of respondents on organisational and logistical matters.

Both criteria were fulfilled, as further described.

### Proficiency: knowledge gain of participants ("test")

Learning evaluation questionnaire with 10 questions was prepared and submitted to the participants at the end of the training session. It aimed at assessing the impact of the training by measuring the participants' understanding of the subjects covered during the training.

The test contained ten multiple-choice questions. The questions were focused on the EU legal framework (crisis management, serious cross-border threats to health, contingency plans for food and feed), the EU RASFF (Rapid Alert System for Food and Feed), the EU EWRS (Early Warning and Response System), investigation and control of foodborne disease outbreaks, emergency preparedness, risk communication.

The results of the tests are summarized in Table 1. A total of 23 participants (95%) successfully passed the test, and 1 participant (5%) failed to respond correctly to the minimum number of questions as per the defined criteria.

Table 1: Results of the knowledge test

| | Questions | | | | | | | | | | Tot | % |
|---|---|---|---|---|---|---|---|---|---|---|---|---|
| ***ID N.*** | **1** | **2** | **3** | **4** | **5** | **6** | **7** | **8** | **9** | **10** | | |
| **1** | 0 | 0 | 1 | 1 | 1 | 1 | 1 | 1 | 0 | 1 | **7** | **70%** |
| **2** | 0 | 0 | 1 | 1 | 1 | 1 | 1 | 1 | 0 | 1 | **7** | **70%** |
| **3** | 1 | 0 | 1 | 1 | 1 | 1 | 1 | 0 | 0 | 1 | **7** | **70%** |
| **4** | 1 | 1 | 1 | 1 | 1 | 0 | 1 | 1 | 1 | 1 | **9** | **90%** |
| **5** | 1 | 1 | 1 | 1 | 1 | 1 | 0 | 1 | 1 | 1 | **9** | **90%** |
| **6** | 1 | 1 | 1 | 1 | 1 | 0 | 0 | 0 | 1 | 1 | **7** | **70%** |
| **7** | 1 | 1 | 1 | 1 | 1 | 1 | 1 | 0 | 0 | 1 | **8** | **80%** |
| **8** | 1 | 0 | 1 | 1 | 1 | 1 | 1 | 1 | 0 | 1 | **8** | **80%** |
| **9** | 1 | 1 | 1 | 0 | 1 | 1 | 1 | 0 | 0 | 1 | **7** | **70%** |
| **10** | 1 | 0 | 1 | 1 | 1 | 0 | 1 | 0 | 1 | 1 | **7** | **70%** |
| **11** | 1 | 1 | 1 | 1 | 1 | 1 | 1 | 1 | 1 | 1 | **10** | **100%** |
| **12** | 1 | 1 | 1 | 0 | 1 | 0 | 1 | 1 | 0 | 1 | **7** | **70%** |
| **13** | 1 | 1 | 1 | 1 | 1 | 1 | 1 | 0 | 0 | 1 | **8** | **80%** |
| **14** | 1 | 1 | 1 | 0 | 1 | 0 | 1 | 1 | 0 | 1 | **7** | **70%** |
| **15** | 1 | 1 | 1 | 1 | 1 | 1 | 1 | 1 | 1 | 1 | **10** | **100%** |
| **16** | 1 | 1 | 1 | 1 | 1 | 1 | 1 | 1 | 1 | 1 | **10** | **100%** |
| **17** | 0 | 1 | 1 | 1 | 1 | 1 | 1 | 0 | 1 | 1 | **8** | **80%** |
| **18** | 1 | 1 | 1 | 1 | 1 | 1 | 1 | 0 | 1 | 1 | **9** | **90%** |
| **19** | 1 | 1 | 1 | 1 | 1 | 1 | 1 | 0 | 1 | 1 | **9** | **90%** |

| | | | | | | | | | | | | |
|---|---|---|---|---|---|---|---|---|---|---|---|---|
| **20** | 1 | 1 | 1 | 1 | 1 | 1 | 1 | 1 | 1 | 1 | **10** | **100%** |
| **21** | 1 | 1 | 1 | 1 | 1 | 1 | 1 | 1 | 1 | 1 | **10** | **100%** |
| **22** | 1 | 1 | 1 | 0 | 1 | 0 | 1 | 1 | 0 | 0 | **6** | **60%** |
| **23** | 1 | 1 | 1 | 1 | 1 | 1 | 0 | 1 | 1 | 1 | **9** | **90%** |
| **24** | 0 | 0 | 0 | 0 | 0 | 0 | 0 | 0 | 0 | 0 | 0 | **0%** |
| **TOT** | *20* | *18* | *23* | *19* | *23* | *17* | *20* | *14* | *13* | *22* | *189* | |

Points assignment. Correct answer: 1; Wrong answer: 0

As can be seen in Table 1, questions 8 and 9 obtained the lowest score of correct answers (respectively 61% and 57%).

Figure 1: Number of correct responses per participants

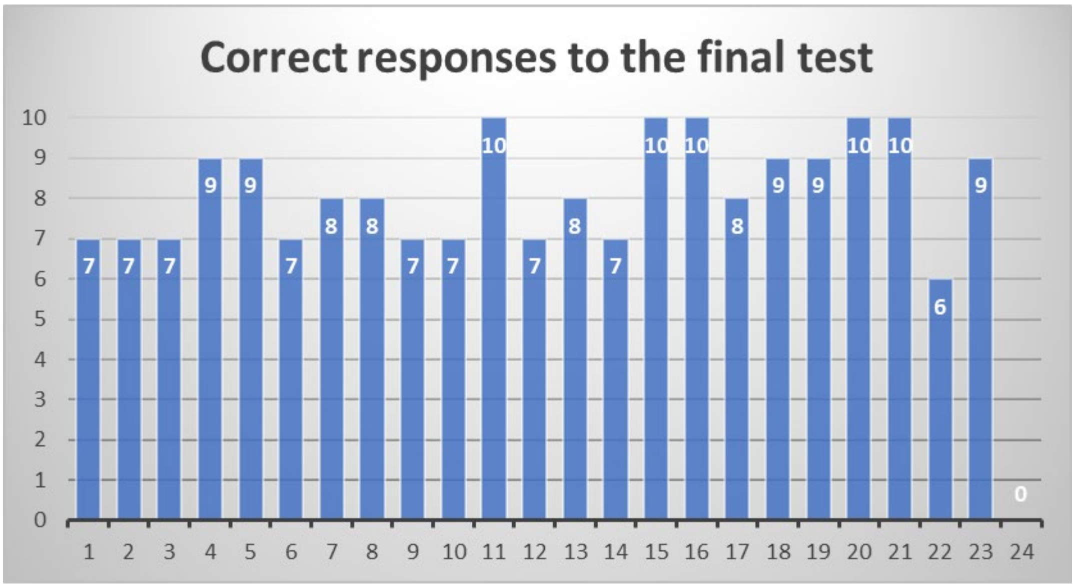

Table 2: Table 2 shows detailed breakdown on the number of participants and the correct responses to 6 or more questions. Percentage of correct answers and relevant number of participants

| N. of correct answers | % of correct answers | N. participants |
|---|---|---|
| 10/10 | 100% | 5 |
| 9/10 | 90% | 5 |
| 8/10 | 80% | 4 |
| 7/10 | 70% | 8 |
| 6/10 | 60% | 1 |
| 5 or less/10 | 0% | 1 |

### Quality of the workshop and participants satisfaction

The quality of the workshop was assessed using a specific questionnaire designed to give the participants the opportunity to make an overall evaluation of the course and to express their opinion and observations.

The first part of the questionnaire gave the participants the possibility to score technical and logistical aspects of the workshop, focusing on effectiveness, methods, materials and facilitators, participants’ contribution, practical application, course length, organization.

A scoring of 0 and 1 represented a negative evaluation.

A scoring of 2 and 3 represented a medium level of satisfaction.

A scoring of 4 and 5 represented a full satisfaction.

Participants scored as summarized at table 3.

Table 3: Results of satisfaction questionnaire

| | | **Participants** | | | | | | | | | | | | | | | | | | | | | **average** |
|---|---|---|---|---|---|---|---|---|---|---|---|---|---|---|---|---|---|---|---|---|---|---|---|
| | | **1** | **2** | **3** | **4** | **5** | **6** | **7** | **8** | **9** | **10** | **11** | **12** | **13** | **14** | **15** | **16** | **17** | **18** | **19** | **20** | **21** | |
| 1 | Fulfilment of expectations | 5 | 5 | 4 | 5 | 5 | 5 | 5 | 5 | 5 | 4 | 5 | 5 | 5 | 4 | 5 | 5 | 5 | 3 | 5 | 5 | 5 | **4,8** |
| 2 | Technical content | 4 | 5 | 5 | 5 | 5 | 3 | 4 | 4 | 4 | 4 | 5 | 5 | 4 | 4 | 5 | 5 | 4 | 3 | 5 | 4 | 5 | **4,4** |
| 3 | Balance between theory/ practice | 5 | 5 | 5 | 5 | 5 | 5 | 4 | 4 | 4 | 4 | 5 | 5 | 5 | 5 | 5 | 5 | 4 | 3 | 5 | 4 | 5 | **4,6** |
| 4 | Methods | 5 | 4 | 4 | 5 | 5 | 5 | 5 | 5 | 5 | 4 | 5 | 5 | 5 | 5 | 5 | 5 | 4 | 5 | 5 | 5 | 5 | **4,8** |
| 5 | Materials | 5 | 2 | 5 | 5 | 5 | 5 | 5 | 4 | 5 | 3 | 5 | 5 | 5 | 5 | 5 | 4 | 5 | 5 | 5 | 5 | 5 | **4,7** |
| 6 | Participants’ contribution | 5 | 4 | 4 | 4 | 5 | 5 | 4 | 5 | 5 | 4 | 5 | 5 | 4 | 5 | 5 | 4 | 5 | 5 | 5 | 4 | 5 | **4,6** |
| 7 | Relevance | 5 | 5 | 4 | 5 | 5 | 5 | 4 | 3 | 3 | 4 | 5 | 5 | 5 | 4 | 5 | 4 | 5 | | 5 | 4 | 5 | **4,5** |
| 8 | Facilitators | 5 | 5 | 5 | 5 | 5 | 5 | 5 | 4 | 5 | 4 | 5 | 5 | 5 | 5 | 5 | 5 | 5 | 5 | 5 | 4 | 5 | **4,9** |
| 9 | Time allocation | 5 | 4 | 4 | 4 | 5 | 5 | 5 | 4 | 4 | 4 | 5 | 5 | 4 | 4 | 5 | 4 | 3 | 5 | 5 | 4 | 5 | **4,4** |
| 10 | Organisation | 5 | 4 | 4 | 5 | 5 | 4 | 5 | 4 | 4 | 4 | 5 | 5 | 4 | 3 | 5 | 5 | 5 | 4 | 5 | 5 | 5 | **4,5** |
| Average | | 4,9 | 4,3 | 4,4 | 4,8 | 5 | 4,7 | 4,6 | 4,2 | 4,4 | 3,9 | 5 | 5 | 4,6 | 4,4 | 5 | 4,6 | 4,5 | 4,2 | 5 | 4,4 | 5 | **4,6** |

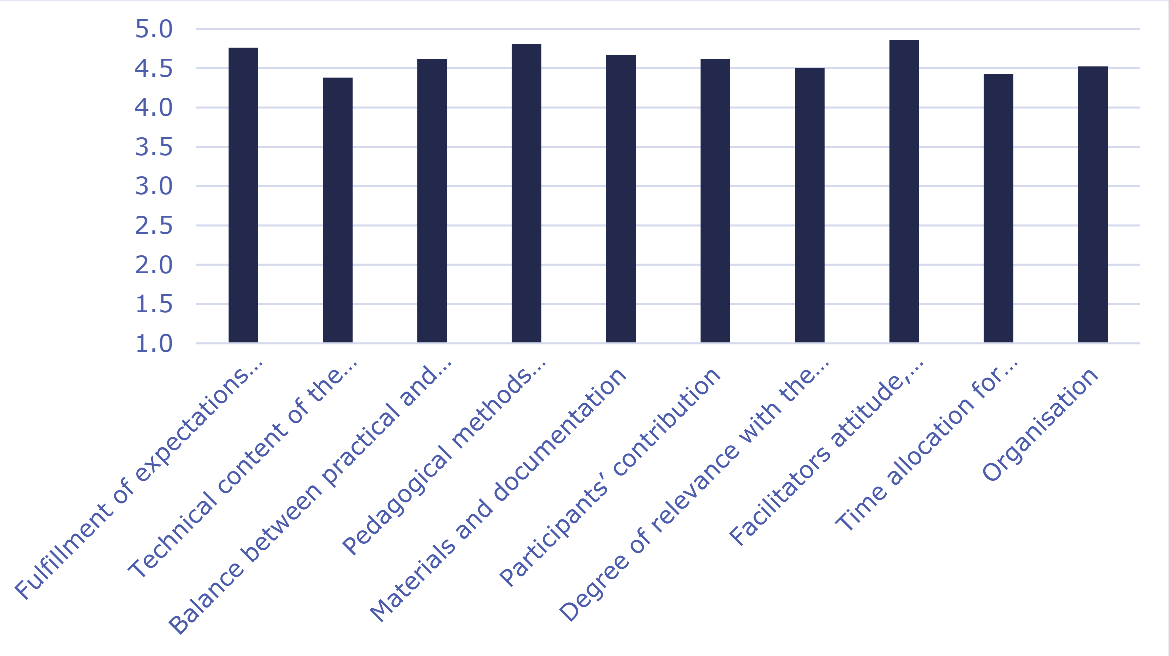

Figure 2: Average scorings provided by participants.

Concerning participants satisfaction, the specific evaluation of "Organisation" (Q10) in the Course evaluation questionnaire obtained 4,5 score on average, and 100% participants gave a positive score.

In the second section of the questionnaire, the participants were asked to express their comments and suggestions. Here some comments from participants are summarized:

- Facilitators were very helpful, advising, pointing out the important points.
- I am looking forward sharing and gaining knowledge.
- Nice workshop; next time maybe lunch to be in the workshop location.
- Everything was excellent in the workshop.
- Maybe to have more days for the exercise.
- We learned a lot throughout the sessions and the exercises were very helpful. I would recommend to everyone.
- I do not recommend to held online this kind of course because communication and coordination between colleague is better and easy than online.

Comments

- In general, answers provided by the participants were highly satisfactory. More in details, the overall average evaluation scored 4,6. Only one participant scored below 4,0.
- The highest score was given to facilitators attitude, preparation and availability, followed by fulfilment of expectations and pedagogical methods. The lowest score (however still at 4,4) was given to technical content and time allocation for presentations and exercises.
- The workshop, as it was planned, was considered to be effective and to achieve the objective (see scoring at question 1).

- Selection of participants was appropriate, considering the scoring of question 7 (degree of relevance with everyday work).
- The opinion regarding the way the exercise was conducted and the pedagogical methods adopted was highly positive (see scoring of questions 4 and 8). This is a favourable signal regarding adopted pedagogical methods and facilitator's attitude and competence.
- The organisation was more than satisfactory. This was due, in large part, to the effort of the local organizer, Mr. Dalibor Vidacak from the Food Safety Agency of Bosnia and Herzegovina. The University of Sarajevo, Center for interdisciplinary studies, provided accommodation and technical equipment for the workshop.

### Conclusion

The overall objectives of the training were met:

- increase IPA countries' knowledge and understanding of general crisis handling concepts and specifically of EFSA's crisis response procedures;
- improve preparedness and response planning for crisis situations in the domain of biological hazards through testing procedures in place at national (contingency plans) and EU level (e.g. the crisis general plan) and crisis handling procedures, helping IPA countries address crisis-preparedness needs and reduce time for action;
- improve coherence, interoperability and coordination at local level and between the local, national, regional and EU levels, to be prepared for communication and decision-making challenges in crisis situations.

Feedback on the event was very positive, with participants giving very high scores on fulfilment of expectations, balance between theoretical and practical parts, pedagogical methods.

The scenario proved to be a valuable tool for learning, well understood by participants and easy enough to follow by the majority of attendants. During the whole workshop, the participants showed a proactive participation and a very good level of interest. The times allocated in the agenda worked well allowing participants to understand the scenario and for their participation.

Taking into account the interest shown by participants, their proactive participation and their feedback at the end of the workshop, it can be concluded that the program was appropriately prepared and that the training session has been carried out properly.

### Recommendations

#### Recommendations for future crisis preparedness activities

- Considering the specificity of the workshop and the required active involvement of all participant to the simulation exercise, with group discussions on the injects, it is recommended that all participants attend the future workshops in physical mode..

- Considering the time constraints in the agenda, and the need to dedicate a large part of the workshop to the simulation exercise, the presentation on the EU legal framework on crisis management coordination and response in the food and feed sectors was provided to the participants before the workshop, as a mean of preparation. However, the facilitators noticed that some participants lacked the necessary knowledge on this topic. Therefore, it is suggested that, in future events, this presentation should be given during the workshop.
- During the simulation exercise, individual rooms/offices should be available for each of the working groups. It will ensure the participants to better concentrate to the exercises and have uninterrupted and more clear communication and interaction within the working groups.

### Recommendations for IPA countries

During the simulation exercise, participants expressed their opinion that there is a need to improve outbreak preparedness and management and to strengthen investigation and reporting of national and multinational food-borne outbreaks. An effective foodborne outbreak management is achieved with a multi-sectoral and multidisciplinary endeavour: the investigation and control of foodborne disease outbreaks requires appropriate skills in the area of public health, environmental health, clinical microbiology, food and veterinary microbiology, food safety, food control, risk assessment and management.

Outbreak preparedness should enable to prevent or limit the extent of an outbreak. Elements commonly identified as being important for emergency preparedness included: clear allocation of responsibilities together with a chain of command, specific organisational structures (“Alert Units” or operational groups), sharing of information and its communication to the public, use of peacetime structures, use of rapid alert systems, early detection, availability of comparable data, availability of risk assessment by independent expert services, a clear definition of alert levels and criteria for their escalation as well as continuous improvement on contingency planning.

Emergency preparedness requires an established set of structures, procedures and coordination mechanisms that function regularly during peacetime, and are escalated during emergencies. The roles of the competent authorities must be well defined, and they should avail necessary legal powers and resources for managing crises, both at a national and at an international level. Cooperation between the competent authorities at all levels shall be guaranteed and procedures describing cooperation within and between different competent authorities should be in place.

Facilitators had an open exchange with participants about the challenges associated with coordinating the activities of multiple response institutions during an actual or potential food crisis. Participants pinpointed that, in some cases, adequate protocols and/or guidelines are not available, for example protocols for the management of outbreaks of foodborne diseases, Inter-Agency protocols for the management of a food crisis, food safety emergency response plans.

A point of discussion was also the efficiency of the surveillance systems in the different IPA countries, and the capability of the surveillance systems to capture and respond to information and data from different sources. Countries need to have a surveillance system based on data collection from consumers, FBOs, laboratories and official control institutions. Moreover, data on foodborne hazards and diseases should be shared between different sectors across the food chain.

On the basis of the outcomes of the above discussions, the following recommendations can be made and are addressed to the IPA countries.

- To evaluate the performance levels of foodborne outbreaks surveillance systems, given that surveillance is the first component of an outbreak management system. The evaluation to be focused on the identification of the capacity to collect, collate, analyse and disseminate information related to indicator pathogens and diseases. This evaluation can be considered as a prerequisite for the improvement of surveillance systems to early detect foodborne outbreaks and promptly find the food in question through epidemiological investigations.
- To map which relevant institutions in the respective countries are involved in the functions/activities that were taken in consideration during the simulation exercise, in order to determine whether all functions are covered and assigned effectively to the different institutions.
- To evaluate whether ordinary effective communication flows and mechanisms exist between the various institutions mentioned in the previous point or whether, vice versa, such communication mechanisms must be activated specifically in the event of food incident because they are not in place in peacetime.
- Where not available, IPA countries are recommended to develop and adopt documented control plan for the management of foodborne outbreaks, specifying roles and responsibilities of all relevant agencies. Objectives of this document should be: to provide a practical resource for professionals involved in foodborne outbreak management, to provide a standardised approach to the management of foodborne outbreaks in the country, to provide a structure for outbreak preparedness at a local and national level, to define the roles of those involved in an outbreak control team. This document should be drafted by a multidisciplinary group, including medical public health specialists, epidemiologists, health officers, laboratory specialists and microbiologists, veterinary and agricultural inspectors, and other professionals as necessary.
- Where not available, IPA countries are recommended to develop and adopt protocols on the management of food incidents. Responding to a food crisis or emergency requires collaboration and co-operation amongst many agencies: these protocols should be drafted by a multi-agency and multidisciplinary team, the aim is to set down the structures and arrangements for inter-agency coordination, communication and operation during the response to a food crisis or potential crisis at national or international level.
- To organize simulation exercises at local and national level, on the basis of the manuals and protocols developed according to the previous bullet points.

## Abbreviations

| | |
|---|---|
| CA | Competent Authority |
| DG SANTE | Directorate-General for Health and Food Safety |
| EC | European Commission |
| ECDC | European Centre for Disease Prevention & Control |
| EFSA | European Food Safety Authority |
| EpiPulse | European surveillance portal for infectious diseases |
| EU | European Union |
| EWRS | Early Warning & Response System |
| IPA | Instrument for Pre-accession Assistance |
| MS | Member State |
| RASFF | Rapid Alert System for Food and Feed |
| SOP | Standard Operating Procedure |
| ROA | Rapid Outbreak Assessment |
| WGS | Whole Genome Sequencing |
